# Coenzyme Q_10_ Supplementation for the Reduction of Oxidative Stress: Clinical Implications in the Treatment of Chronic Diseases

**DOI:** 10.3390/ijms21217870

**Published:** 2020-10-23

**Authors:** Francisco Miguel Gutierrez-Mariscal, Antonio Pablo Arenas-de Larriva, Laura Limia-Perez, Juan Luis Romero-Cabrera, Elena Maria Yubero-Serrano, Jose López-Miranda

**Affiliations:** 1 Unidad de Gestión Clinica Medicina Interna, Lipids and Atherosclerosis Unit, Maimonides Institute for Biomedical Research in Córdoba, Reina Sofia University Hospital, University of Córdoba, 14004 Córdoba, Spain; fmgutierrezm@hotmail.com (F.M.G.-M.); aparenaslarriva@gmail.com (A.P.A.-d.L.); laura_limia@hotmail.com (L.L.-P.); juanluroca855@gmail.com (J.L.R.-C.); 2 CIBER Physiopathology of Obesity and Nutrition (CIBEROBN), Institute of Health Carlos III, 28029 Madrid, Spain

**Keywords:** Coenzyme Q_10_, ubiquinone, oxidative stress, antioxidant capacity, cardiovascular risk factors, cardiovascular disease, kidney disease, non-alcoholic fatty liver disease, chronic obstructive pulmonary disease, neurodegenerative diseases

## Abstract

Apart from its main function in the mitochondria as a key element in electron transport, Coenzyme Q_10_ (CoQ_10_) has been described as having multiple functions, such as oxidant action in the generation of signals and the control of membrane structure and phospholipid and cellular redox status. Among these, the most relevant and most frequently studied function is the potent antioxidant capability of its coexistent redox forms. Different clinical trials have investigated the effect of CoQ_10_ supplementation and its ability to reduce oxidative stress. In this review, we focused on recent advances in CoQ_10_ supplementation, its role as an antioxidant, and the clinical implications that this entails in the treatment of chronic diseases, in particular cardiovascular diseases, kidney disease, chronic obstructive pulmonary disease, non-alcoholic fatty liver disease, and neurodegenerative diseases. As an antioxidant, CoQ_10_ has proved to be of potential use as a treatment in diseases in which oxidative stress is a hallmark, and beneficial effects of CoQ_10_ have been reported in the treatment of chronic diseases. However, it is crucial to reach a consensus on the optimal dose and the use of different formulations, which vary from ubiquinol or ubiquinone Ubisol-Q_10_ or Qter^®^, to new analogues such as MitoQ, before we can draw a clear conclusion about its clinical use. In addition, a major effort must be made to demonstrate its beneficial effects in clinical trials, with a view to making the implementation of CoQ_10_ possible in clinical practice.

## 1. Introduction

Coenzyme Q_10_ (CoQ_10_) is a lipid-soluble and biologically active quinone which comprises a benzoquinone ring with an isoprenoid side-chain. Festenstein et al., 1955 and Crane et al., 1957 isolated and characterized this compound for the first time and established its function as an electron carrier in the mitochondrial electron transport chain [1,2]. Apart from its principal function in the mitochondria, CoQ_10_ has been described as having multiple functions, such as oxidant action in signal generation and controlling the cellular redox state, a role in proton gradient formation in the endomembrane and the plasma membrane, and helping to control membrane structure and phospholipid status [3,4], among others. For a complete, in-depth analysis of the biological and physiological functions of CoQ_10_, see Gutierrez-Mariscal et al., 2018 [5].

**Figure 1:**
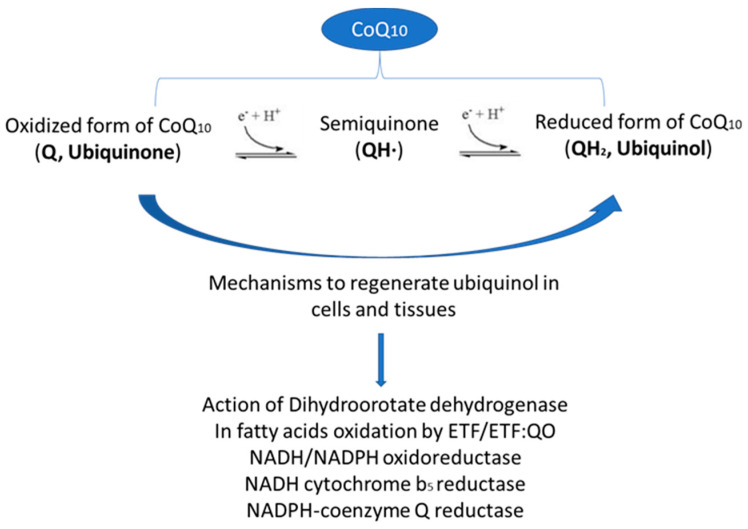
Coenzyme Q_10_ redox forms and mechanisms in cells for the recovery of the active antioxidant form, ubiquinol. CoQ_10_: Coenzyme Q_10_; ETF/ETF: QO: electron transfer flavoprotein/electron transfer flavoprotein:ubiquinone oxidoreductase; NADH/NADPH: nicotinamide adenine dinucleotide reduced/nicotinamide adenine dinucleotide phosphate.

Among all the functions attributed to CoQ_10_ mentioned above, the most important and relevant of its actions is the potent antioxidant capability of its coexistent redox forms (ubiquinone, semi-ubiquinone, and ubiquinol), which act in the mitochondrial membrane, as well as in other membranes in the cell and in the plasma and cytoplasm. These antioxidant properties in the electron transport chain in the mitochondria enhance the efficiency of the electron transport, preventing the loss of uncontrolled electrons, help to recycle other antioxidants such as vitamin C or vitamin E, and directly act on free radicals or oxidants, reducing and neutralizing the compounds. The capability of CoQ_10_ to exchange electrons one-by-one as it converts between the three redox forms makes these antioxidant actions possible. Presumably, the reduced form of CoQ_10_, ubiquinol, is the active agent involved in the antioxidant function, so the cells and tissues must have molecular mechanisms by which they recover their active form. Briefly, these mechanisms include the action of dihydroorotate dehydrogenase in the inner mitochondrial membrane, which is involved in pyrimidine biosynthesis and reduces ubiquinone by the oxidation of dihydroorotate to orotate, and the involvement of CoQ_10_ in the flavoprotein/electron transfer of flavoprotein:ubiquinone in the oxidoreductase system, which enables the ubiquinol to recover by participating in the oxidation of the fatty acids. Here, CoQ_10_ is kept in equilibrium between its redox forms outside the mitochondria by the actions of three enzymes: nicotinamide adenine dinucleotide reduced/nicotinamide adenine dinucleotide phosphate (NADH/NADPH) reduced oxidoreductase, NADH cytochrome *b_5_* reductase, and NADPH-coenzyme Q reductase (Figure 1) [6,7,8]. CoQ_10_ has a ubiquitous location and is present in all the membranes of the cell. The major source of CoQ_10_ comes from its endogenous biosynthesis, which occurs in all the tissues of an organism, although a minor proportion of CoQ_10_ derives from dietary sources. However, from a clinical point of view, it is important to note that endogenous production deficiency has been reported in the pathophysiology of different diseases, and that aging is associated with a major level of decay. 

**Figure 2:**
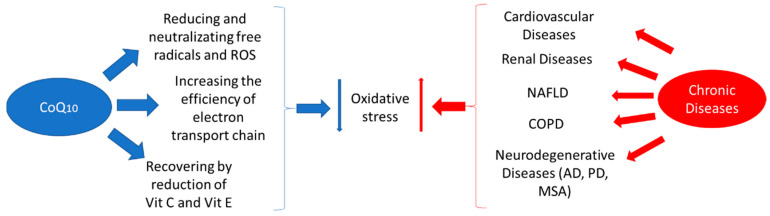
Coenzyme Q_10_ mechanisms against oxidative stress associated with chronic disease. CoQ_10_: Coenzyme Q_10_; Vit C: vitamin C; Vit E: vitamin E; NAFLD: non-alcoholic fatty liver disease; COPD: chronic obstructive pulmonary disease; AD: Alzheimer’s disease; PD: Parkinson’s disease; MSA: multiple system atrophy.

In most of the studies carried out on the effects or uses of CoQ_10_ supplementation, the main benefit comes from its ability to reduce oxidative stress, which is the most important, most relevant, and most commonly studied function described for this compound. For this reason, our objective was to review the latest publications on the effects of CoQ_10_ supplementation against the oxidative stress in chronic disease (Figure 2). In this review, we aimed to explore the influence of CoQ_10_ on diseases less commonly reviewed in the literature, such as chronic obstructive pulmonary disease and non-alcoholic fatty liver disease, as well as to extensively update our knowledge regarding the latest findings concerning new formulations of CoQ_10_ in the study of neurodegenerative diseases. We also aimed to explore and analyse our current knowledge of this issue in other diseases such as cardiovascular and renal diseases, and its future perspectives.

## 2. Biology of CoQ_10_

The biosynthesis of CoQ_10_ in an organism is carried out by a pathway involving at least 11 genes, known as *COQ* genes, which are well conserved between species [9]. The first step in the *de novo* cellular synthesis of CoQ_10_ consists of the synthesis of the benzoquinone ring, initiated with 4-hydroxybenzoate together with the side chain precursor, acetyl-CoA [10,11]. One remarkable event in CoQ_10_ biosynthesis is the common pathway it shares with cholesterol biosynthesis. This fact implicates that the use of statins in the treatment of hypercholesterolemia goes along with a parallel reduction in the synthesis of CoQ_10_.

Wherever CoQ_10_ comes from, whether from endogenous or external sources, it is found widely in all subcellular compartments. There must be a regulated mechanism for its distribution from the mitochondria to other membranes: for instance, an endomembrane system has been reported to be involved in the transport of CoQ_10_ in plasma membranes [12,13]. Furthermore, CoQ_10_ transport must also occur in the opposite direction, from the exogenous sources to the intracellular compartments and via the plasma membrane to the rest of organelle membranes, including the inner mitochondria membrane. However, the subcellular distribution of CoQ_10_ is asymmetrical, since most of this quinone is located in the inner mitochondrial membrane (40–50%), and to a lesser extent in other organelles such as the Golgi, endoplasmic reticulum or lysosomes. This fact is mainly a reflection of its principal function as an electron carrier in the mitochondria. 

Regarding its distribution in the organisms, it has been reported that it is produced by all cells, in varying amounts between different organs. Human CoQ_10_ levels of 8 μg/g can be found in the lung and up to 114 μg/g in the heart. The content of CoQ_10_ in the organs depends on the energy requirements or metabolic activity, and so tissues with high concentrations of this compound include the heart, kidney, liver, and muscle, mainly [14]. Moreover, the requirements of the oxidative metabolism seem to be the key factor in the regulation of the varying content of CoQ_10_ among different tissues [15].

The levels of CoQ_10_ found in cells and tissues must be highly regulated and depend on age, race, sex, and dietary conditions [16]. Remarkably, CoQ_10_ levels are disrupted by ill health and disease status, and in this regard, Alzheimer’s disease, cardiomyopathies, and diabetes patients present lower levels of CoQ_10_ compared to healthy people [16,17]. 

The uptake and distribution of CoQ_10_ after oral ingestion are determined by its lipophilic characteristics, which give it extremely low solubility in water. In this sense, the typical regimen for oral administration of CoQ_10_ takes advantage of its lipophilic solubility and recommends co-administration with lipid-rich foods [18]. Despite these recommendations, research into exogenous CoQ_10_ absorption and bioavailability has proved high variability that depends on the type of CoQ_10_ preparation studied [15,19]. Many formulations have been developed to improve CoQ_10_ solubility in the human body. Recent new formulations for CoQ_10_ are based on enhancing its water-solubility, as found in Qter^®^ or Ubisol-Q_10_. Efforts have also been made to discover new formulations or analogues of CoQ_10_ with greater solubility and antioxidant effects, such as MitoQ. Regarding adverse effects of CoQ_10_ supplementation, there exists no important adverse effect described in the bibliography. The exogenous uptake of this quinone has been reported to interfere with endogenous biosynthesis, and no plasma or tissue accumulation has been described. Villalba et al., reported that doses up to 3000 mg/day did not produce serious adverse effects, but nausea and other gastrointestinal effects were reported [19].

To date, the information about an optimal endogenous therapeutic range for CoQ_10_ after exogenous supplementation is controversial, suggesting oral supplementation doses up to 2400 mg per day in adults and up to 30 mg/kg daily in children, divided into three doses per day in patients with CoQ_10_ deficiency [20]. Although there is no evidence of therapeutic CoQ_10_ concentrations in patients with CoQ_10_ deficiency, a study performed in patients with congestive heart failure found that a blood CoQ_10_ concentration of 4.1 μM was therapeutically effective. Different studies have suggested that blood mononuclear cells may be a better tissue for monitoring endogenous CoQ_10_ than plasma, since they found a strong correlation between skeletal muscle and blood mononuclear cell CoQ_10_ status, but not with plasma in patients with no evidence of mitochondrial function-related diseases [21,22].

## 3. Methodology of Review

For the purpose of this review, we performed a search in Pubmed on 6 June 2020 using the key words “Coenzyme Q_10_ supplementation oxidative stress chronic disease”. The outcome of the search showed 38 publications between 2001–2019, which included eight reviews, 10 clinical trials, and three meta-analyses. Since we recently performed an extensive clinical review of CoQ_10_ supplementation published in 2018 [5], we focused on recent advances from 2018 to the present day and different issues or diseases not included in that review. After revisiting this bibliography, we decided to include the recent publication of CoQ_10_ supplementation in cardiovascular diseases, kidney diseases, chronic obstructive pulmonary disease (COPD), non-alcoholic fatty liver disease (NAFLD), and neurodegenerative diseases. The selection was focused on the evidence of involvement of oxidative stress in their aetiology because of an overproduction of reactive oxygen species (ROS) due to metabolic conditions of the diseases or by a CoQ_10_ deficiency associated with the disease. For each of these diseases, we performed a new search on Pubmed using “CoQ_10_/coenzyme Q_10_/ubiquinone supplementation cardiovascular disease/heart failure/coronary artery disease/kidney disease/kidney failure/end-stage renal disease/nephrotic syndrome/chronic obstructive pulmonary disease/NAFLD/neurodegenerative disease/Alzheimer’s disease/Parkinson’s disease/multiple system atrophy oxidative stress”. From the publications obtained in these searches, we selected those with greater relevance and a more recent publication date.

## 4. CoQ_10_ and Cardiovascular Risk Factors

In light of current evidence, the clinical impact of CoQ_10_ supplementation on CVD through the reduction of cardiovascular risk factors has been analysed with the aim of improving patient health and quality of life. 

### 4.1. Dyslipidaemias 

Dyslipidaemia, particularly in the context of high levels of low-density lipoprotein (LDL)-cholesterol and low levels of high-density lipoprotein (HDL)-cholesterol, induces mitochondrial dysfunction, which in turn causes oxidative stress by the overproduction of ROS. The evidence indicates that increased levels of ROS can trigger the development of endothelial dysfunction and inflammation [23]. Although the effect of CoQ_10_ supplementation on plasma lipid levels is quantitatively small, different meta-analysis and systematic reviews have supported beneficial effects in a range of different patient profiles. In a recent meta-analysis conducted by Sharifi et al. [24], CoQ_10_ administration significantly reduced triglyceride (TG) concentrations in patients with metabolic disease. Another meta-analysis including six clinical trials suggests that CoQ_10_ could mildly reduce the lipoprotein (a) plasma (Lp(a)) levels in patients with Lp(a) ≥ 30 mg/dL, although no other plasma lipids such as total cholesterol, LDL-cholesterol, HDL-cholesterol or TG [25] were affected. However, a recent systematic review performed in patients with coronary artery disease (CAD) found that CoQ_10_ supplementation significantly decreased total cholesterol and increased HDL-cholesterol levels, but did not affect TG, LDL-cholesterol, and Lp(a) levels [26]. In this context, it is important to mention that cholesterol biosynthesis and CoQ_10_ share a common initial pathway (the mevalonate pathway). In patients with CAD and heart failure, treatment with statins may lower the level of CoQ_10_ [27]. Here, co-administration of CoQ_10_ and statin therapy is recommended to avoid myopathic side effects, as well as to enhance antioxidant enzyme activities and lower inflammation in this type of patient [28,29].

Different mechanisms, by which CoQ_10_ supplements could modulate circulating lipid profiles, have been proposed. Lee et al. found that CoQ_10_ increased the fatty acid oxidation through AMPK-mediated peroxisome proliferator-activated receptor-γ(PPAR-γ) induction in 3T3-L1 preadipocytes [30]. Moreover, CoQ_10_ could also act to suppress oxidized LDL-induced endothelial oxidative injuries by the modulation of oxidized low-density lipoprotein receptor 1-mediated ROS generation via the AMP-activated protein kinase/Protein kinase C/NADPH oxidase signalling pathway [31]. PPAR-γ, a nuclear receptor protein which acts as a ligand-activated transcription factor, has been fully described as playing a role in regulating gene expression related to insulin and lipid metabolism, differentiation, proliferation, survival, and inflammation [32].

### 4.2. Hypertension

Hypertension is a key risk factor for almost all the different cardiovascular diseases. Although many pharmacological treatments have shown an optimal efficacy in lowering blood pressure and in modestly decreasing cardiovascular mortality, hypertension remains highly prevalent, especially in the elderly [33]. 

The effect of CoQ_10_ on blood pressure has been investigated, as an alternative treatment, in several controlled intervention studies in human subjects. CoQ_10_ exerts a direct effect on the endothelium, improving vascular smooth muscle activity, and reducing vasoconstriction and blood pressure [34]. In fact, the protective effect of CoQ_10_ supplementation on hypertension is associated with its ability to prevent oxidative/nitrative stress and reduce inflammation, which results in a recoupling of endothelial nitric oxide synthase (eNOS) [35]. The beneficial effects of CoQ_10_ on blood pressure have been tested in a range of 100 mg to 200 mg/day through different controlled intervention studies in human subjects [36,37,38]. Here, CoQ_10_ produced a decrease in systolic blood pressure (between 11–17 mmHg) and a reduction of 8 mmHg in diastolic blood pressure, thus demonstrating the possible role of CoQ_10_ as a hypotensive agent with and without being combined with other conventional anti-hypertensive therapies. In fact, Zhang et al. reported that CoQ_10_ modulates the angiotensin effect in sodium retention and decreases the level of aldosterone [39]. However, due a degree of controversy regarding the effects of CoQ_10_ in lowering blood pressure, more well-controlled clinical trials are required to investigate this potential property of CoQ_10_.

### 4.3. Endothelial Dysfunction

Endothelial dysfunction, a condition that contributes to the pathogenesis of vascular disease in T2DM, is considered a reliable marker of subclinical atherosclerotic cardiovascular disease, as it appears before the development of atherosclerotic lesions or the occurrence of clinical events. Endothelial dysfunction is also described as being involved in the deterioration of cardiac function, especially in conditions of metabolic disease [40]. Several studies have demonstrated the effect of CoQ_10_ supplementation on endothelial function in patients with type 2 diabetes mellitus, CAD or in elderly people [41,42,43], showing that flow-mediated dilation (FMD) or nitroglycerin-mediated dilation (NMD) and extracellular superoxide dismutase activity increased in most of the subjects treated with CoQ_10_, which could be attributed to its antioxidant and anti-inflammatory activity [36,43,44]. This effect of CoQ_10_ results in reduced levels of oxidative stress markers such as advanced glycation end products [45] and a decreased rate of inactivation of NO to peroxynitrite by superoxide radicals that could improve vascular tone as well as endothelial function. On the other hand, in vitro studies pointed out that CoQ_10_ can efficiently prevent high glucose-induced endothelial cell apoptosis [46] and increase endothelial progenitor cells angiogenesis through a mechanism involving AMPK, eNOS, and heme oxygenase-1 pathways [31].

## 5. CoQ_10_ and Cardiovascular Diseases 

Different studies have analysed the effect of CoQ_10_ supplementation on CVD, with a view to evaluate its use as a therapeutic approach in the reduction of clinical CVD manifestations. Here, we report the main conclusions on CoQ_10_ supplementation and two of the most commonly studied CVD manifestations: 

### 5.1. Heart Failure and Coronary Heart Disease

Heart failure (HF) is a global epidemic in health care and a leading cause of mortality and morbidity in both Western and Eastern societies [47]. Despite the prevention and treatment of HF and the development of related drugs, mortality rates from HF are higher than 10% per year, even reaching 20% to 50% in some settings [48]. 

Mitochondrial dysfunction is an important characteristic of HF, mainly characterized by a deficit in the production of myocardial adenosine triphosphate, deflection of calcium exchange, and increased production of ROS leading to endothelial dysfunction (see Section 4.3). Moreover, a relative deficiency of CoQ_10_ concentrations has been found in HF patients, suggesting that the depletion of CoQ_10_ is proportional to the reduction of CoQ_10_ myocardial tissue concentrations and to the severity of the disease [49]. Therefore, since CoQ_10_ plays a key role in cell energetics in the mitochondria, acting as an effective anti-inflammatory and antioxidant agent, and also improving endothelial function, it is plausible that CoQ_10_ supplementation has potential benefits as a therapeutic option for HF patients. In this sense, regarding HF and coronary heart disease, the beneficial effects of CoQ_10_ supplementation cannot be only attributable to its antioxidant capacity, but also other properties mentioned above. Several lines of evidence have pointed out the increased CVD risk for South Asians compared to other ethnicities [50]. In fact, Hughes et al. found that Indians showed higher coronary heart disease (CHD) prevalence than Malays or Chinese, with Malays higher than Chinese [51]. Although these authors could not explain this higher susceptibility of Indians to CHD by differences in cardiovascular risk factors, they found reduced levels of plasma ubiquinol and total CoQ_10_ in Indian compared to Chinese males [51], suggesting that differences in oxidative stress status between them may reflect their distinct susceptibility to CHD.

Over the last few years, several clinical studies have investigated the possibility of using CoQ_10_ in the prevention of HF. In one of the most important clinical studies related to this research area, it was shown that the intake of 300 mg of CoQ_10_ in 420 patients with moderate or severe HF resulted in a reduction in the rate of major adverse cardiac events (MACE), cardiovascular mortality, all-cause mortality, and incidence of hospital stays for HF after two years compared to those patients treated with a placebo [52]. This fact has been confirmed in a subsequent meta-analysis of 14 randomized clinical trials (RCTs), including 2149 patients, although no significant differences were observed in the endpoint of left ventricular ejection fraction between the group treated compared to a placebo [53]. However, no additional efficacy was found, after a short-term supplementation of CoQ_10_ in HF patients with preserved ejection fraction, in improving left ventricle diastolic function [54].

Despite the effects of CoQ_10_ in improving the antioxidant systems in HF, in general, the varied trial designs with different follow-up duration, heterogeneous populations, study outcomes, and different administered doses of CoQ_10_ make it difficult to discern a clear effect of CoQ_10_ in HF.

### 5.2. Myocardial Infarction

According to the recent update of its definition, myocardial infarction occurs due to myocardial cell death caused by prolonged ischaemia [55]. In this pathology, myocardial complications appear such as left ventricular dysfunction, which is related to necrosis and loss of functioning in the myocardium and, consequently, pathological remodelling. These processes seem to be related to high oxidative stress, with reperfusion-induced free radical damage, lipid peroxidation, and decreased energy production, in which CoQ_10_ deficiency could play a role [56,57]. In this context, several studies have analysed CoQ_10_ supplementation in this pathology to prevent complications in patients with myocardial infarction. In an RCT, Mohseni et al. found that CoQ_10_ supplementation, at a dose of 200 mg/day for 12 weeks, was able to improve blood pressure and reduced serum HDL-cholesterol as well as LDL-cholesterol/HDL-cholesterol and total cholesterol/HDL-cholesterol ratios after CoQ_10_ supplementation in patients who presented hyperlipidaemia but also myocardial infarction [58]. These results were confirmed in another randomized study, by the same research group, in which CoQ_10_ supplementation increased HDL-cholesterol but also produced a decrease in the inflammatory status (serum ICAM-1 and IL-6 levels) in patients with myocardial infarction [59]. The anti-inflammatory effects of CoQ_10_, but not the improvement of any cardiometabolic marker, were also observed in diabetic patients with CAD, which suggests that diabetes could infer different underlying pathogenetic mechanisms for myocardial infarction [60].

Regarding the possibility of using CoQ_10_ supplementation as an agent which can prevent cardiac remodelling in patients with myocardial infarction, 24-weeks of supplementation of CoQ_10_ (120 mg/day) resulted in the maintenance of the sphericity index and a reduced alteration of the wall thickening abnormality at the infarct site, compared to the placebo, in patients with persistent left ventricular dysfunction [61]. However, long-term RCTs are needed to confirm these preliminary data.

## 6. CoQ_10_ and Chronic Kidney Disease

Chronic kidney disease (CKD) is increasingly considered a global health concern. Patients with CKD have a high risk of CVD-related mortality and morbidity. It is estimated that more than 50% of the mortality in those patient with end-stage renal disease (ESRD) on dialysis is related to CVD and its complications [62]. Moreover, to these data we should add the fact that all the people worldwide who currently receive treatment for dialysis or a kidney transplant to stay alive only represent 10% of people who actually need treatment to live [63]. Patients with CKD and ESRD showed increased levels of oxidative stress due to an imbalance between ROS and antioxidant systems, turned in favour of ROS. It has recently been suggested that the high mortality in this type of patient may be attributable to an increased risk of CVD as a result of increased oxidative stress [64,65]. Moreover, CoQ_10_ levels are reduced in patients with CKD [66], suggesting the possible use of the supplementation of this quinone as an antioxidant therapy in these types of patients.

There are different studies analysing the role of CoQ_10_ supplementation in both metabolic profiles and oxidant/antioxidant status in patients with CKD. In a meta-analysis conducted by Bakhshayeshkaram et al. [64], CoQ_10_ supplementation significantly reduced total cholesterol, LDL-cholesterol, malondialdehyde (MDA), and creatinine levels but had no effect on fasting glucose, insulin homeostatic model assessment of insulin resistance, and C-reactive protein (CRP) concentrations in patients diagnosed with CKD. The effect of the molecular mechanisms involved in CoQ_10_ on metabolic profiles is not clear, although it has been suggested that the ingestion of CoQ_10_ may induce gene expression of PPAR-γ by activating the calcium-mediated AMPK pathway and inhibiting differentiation-induced adipogenesis (see Section 4.1.) [30,31].

As regards the antioxidant properties of CoQ_10_, a randomized, double-blind, placebo-controlled trial showed that CoQ_10_ supplementation (120 mg/day) in CKD patients produced a reduction in the number of patients on dialysis compared with the placebo group after 28 days of treatment [67]. More recently, in a safety study of oral CoQ_10_ administration in hemodialysis patients, the results indicated an dose-dependent effect of CoQ_10_ in the reduction of oxidative stress, which, in turn, improved mitochondrial function and decreased oxidative stress in patients receiving hemodialysis [68]. In a recent double-blind, parallel group, randomized clinical trial, daily supplementation with 1200 mg of CoQ_10_ was proved safe and resulted in a reduction in plasma concentrations of F2-isoprostanes, a marker of lipid oxidation in patients undergoing maintenance hemodialysis [69]. Animal studies demonstrated that long-term CoQ_10_ supplementation increases kidney CoQ_10_ levels sufficiently to rescue hydrogen sulfide (H_2_S) oxidation by increasing sulfide:quinone oxidoreductase (SQOR) levels and thereby preventing renal failure [70]. 

## 7. CoQ_10_ and Chronic Obstructive Pulmonary Diseases 

According to current evidence, oxidative stress is involved in the mechanisms underlying the initiation and progression of respiratory diseases. Epidemiological and clinical studies have demonstrated the potential of pulmonary oxidative stress to increase mortality, since it increases the incidence of respiratory diseases [71]. Chronic obstructive pulmonary disease (COPD) is a common disease, with persistent airflow limitation as the main clinical outcome. The disease is frequently associated with nutritional abnormalities and skeletal muscle dysfunction, contributing to exercise intolerance and poor health status. Regarding its pathophysiology, some authors in the past decade have demonstrated higher values of oxidized CoQ_10_ in patients with COPD compared with healthy controls, suggesting the involvement of oxidative stress in the pathogenesis of the disease [72]. Smoking is considered the most important factor leading to COPD [73]. COPD induced by cigarette smoke extract presents pulmonary vascular cell injury, due to the elevated ROS levels, with pathophysiological effects such as cell apoptosis, inflammation, and endothelial barrier disruption [74]. Few clinical trials have been carried out to study the effects of CoQ_10_ supplementation as an antioxidant counteracting the oxidative stress in the basis of the disease. DeBenedetto et al. demonstrated in a randomized study that 2-months’ supplementation with QTer^®^ (a formulation for CoQ_10_) and creatine significantly improved exercise capacity, body composition, dyspnea, and daily activities, and was associated with positive changes in the plasma metabolic profile in COPD patients on long-term oxygen therapy [75]. However, since the combined use with creatine, it is difficult to attribute all the beneficial outcomes observed by the authors to only the action of QTer^®^, mainly the improvement in the exercise capacity and the daily activities, since creatine contribute to a better metabolism in the muscle function. Most recently, Chen S et al. showed that pre-treatment with the mitochondrial-targeting antioxidant MitoQ, an orally active antioxidant which aims to mimic the role of CoQ_10_ and even augment substantially its antioxidant capacity [76], protected against the endothelial barrier dysfunction induced by smoking. Indeed, MitoQ was also capable of reversing the classic NF-kB signalling pathway, preventing inflammation, by a reduction in mitochondrial damage in HUVEC culture cell [77], illustrating the effect of this compound not only in oxidative stress, but also in reducing inflammation.

## 8. CoQ_10_ and Non-alcoholic Fatty Liver Disease

Non-alcoholic fatty liver disease (NAFLD) has become one of the most important and relevant chronic liver diseases in the world, mainly due to the global obesity pandemic. Despite the clinical, epidemiological, and economic relevance of this disease, to date, there are currently no specific drugs approved for this pathology.

NAFLD is a hepatic manifestation of metabolic syndrome associated with obesity and increased CVD risk, characterized by an increase in insulin resistance and accumulation of large droplets of TG within the hepatocytes [78]. This excess fat in the liver leads to an increase in hepatokine secretion and gluconeogenesis, a decrease in glycogen synthesis, an inhibition of insulin signalling, and chronic inflammation which increases the risk of progressive chronic liver disease with fibrosis, cirrhosis, and an increased risk of hepatocellular carcinoma [79].

Different studies have supported the idea that oxidative stress may be a primary cause of liver fat accumulation in NAFLD [80], in which ROS could play a role in fibrosis development [81]. In fact, it has been demonstrated that patients with NAFLD showed mitochondrial dysfunction with decreased concentrations of antioxidant defenses [82]. In this context, given the important role of CoQ_10_ in mitochondria and its function as an efficient endogenously-synthesized antioxidant in all membranes, it is plausible that CoQ_10_ contributes to the delay in NAFLD development and progression [83,84]. 

Recent research has addressed two main approaches: the study of the relationship between CoQ_10_ metabolism and NAFLD pathology and the influence of dietary supplementation with CoQ_10_ on the development of this disease. CoQ_10_ exerts anti-adipogenic properties and thus could have a positive impact on NAFLD. It is suggested that CoQ_10_ could act as an AMPK activator, regulating hepatic lipid metabolism (suppressing lipogenesis and activating fatty acid oxidation) to inhibit the abnormal accumulation of hepatic lipids and prevent NAFLD progression [85]. CoQ_10_ could also regulate the inflammatory response through nuclear factor kappa B (NF-kB)-dependent gene expression, in that CoQ_10_ deficiency might produce an increase in pro-inflammatory molecules [86,87]. In addition, CoQ_10_ supplementation (100 mg/day), for 12-weeks, in 41 patients with NAFLD, decreased the levels of liver enzymes, such as aspartate aminotransferase (AST) and gamma-glutamyl transpeptidase, markers of systemic inflammation (tumor necrosis factor α and high-sensitivity CRP (hs-CRP)), and increased the levels of adiponectin [88]. In another RCT, in 44 NAFLD patients, CoQ_10_ supplementation (100 mg/day) for four weeks was associated with significantly decreased waist circumference, serum AST, and total antioxidant capacity concentration [89]. However, more clinical studies would be needed to elucidate the mechanisms by which this quinone exerts its beneficial properties in NAFLD.

## 9. CoQ_10_ and Neurodegenerative Diseases or Neural Diseases

Oxidative stress seems to be commonly involved in neurological diseases, mainly in Alzheimer’s disease (AD), Parkinson’s disease (PD), and amyotrophic lateral sclerosis disease. The main consequences of oxidative stress in these diseases result in glutathione loss, oxidative DNA, and protein damage [90,91]. Here, supplementation with CoQ_10_ appears to be a potential treatment for these diseases, thanks to its properties in ameliorating the oxidative stress, as well as mitochondrial functions. It has been fully described that, with aging, a decline in the endogenous biosynthesis of CoQ_10_ takes place, so the supplementation with this compound in AD as well as PD influences the restoration of endogenous levels as well as counteracting oxidative stress. It is important to mention here that regarding supplementation with this compound in these diseases, the diffusion throughout the blood brain barrier (BBB) might be taken into account. In this sense, some authors have described that mitochondrial dysfunction, increased oxidative stress, and chronic brain hypoperfusion contribute to the disruption of the BBB and cause damage to brain parenchymal cells [92,93]. Furthermore, Duberley et al. explained that, although the reason for the refractory nature of the neurological symptoms associated with CoQ_10_ deficiency in response to CoQ_10_ supplementation remain to be clarified, they may include the poor transfer of CoQ_10_ across the BBB as a factor to take into account [94]. However, studies carried out in animal models have shown that when CoQ_10_ is used in large doses, it can be taken up by all tissues, including the brain [15].

### 9.1. Alzheimer’s Disease

Alzheimer’s disease is the most common neurodegenerative disease and the main form of dementia all over the world. AD is mainly characterized by a decline in neurocognitive function leading to severe morbidity and eventually death [95]. The exact aetiology of the disease is not yet understood, but what is known is that certain pathological features comprise the formation of toxic β-amyloid plaques, together with neuro-fibrillary tangles and neuron loss in the hippocampus [96,97]. Behind these physiological and structure presentations of the disease, there are a number of molecular mechanisms described as being associated with AD, such as increased oxidative stress [98], mitochondrial dysfunction, impaired autophagy activity, and a resulting accumulation of defective proteins/organelles [99]. Based on that premise, several authors have suggested the potential beneficial effects that the action of CoQ_10_ could exert on these molecular events occurring in AD, which could ameliorate the symptoms and even reverse its effects on cognitive functions. Unfortunately, few studies exist regarding CoQ_10_ supplementation in human clinical trials in order to investigate the beneficial effects of this antioxidant in the progression of this neurodegenerative disease. However, important advances have been made recently in the study of the pathophysiological consequences of supplementation in animal models, which can serve as a precedent and basis for future studies in humans which could finally clarify the utility of this relevant antioxidant in clinical medicine. 

Most of the in vitro and in animal model studies have failed to progress to human clinical trials, and according to the authors, this fact could be explained by the effective CoQ_10_ levels found in humans. The doses applied in in vitro studies may not correspond with those existing in the in vivo cells due to the poor bioavailability of the oil formulation for CoQ_10_. In this context, new water-soluble formulations such as Qter^®^ or Ubisol-Q_10_ have proved their efficacy in animal models, which could provide a promising starting point to consider them for use in clinical trials in humans. Recent works using the formulation Ubisol-Q_10_ have demonstrated the beneficial effects of its supplementation or administration. Muthukumaran et al. reported that treatment with Ubisol-Q_10_ inhibited Alzheimer’s-type behavioural and pathological symptoms in a double transgenic mouse (TgAPEswe, PSEN1dE9) model of AD [98]. On the other hand, Vegh et al. demonstrated, using transgenic AD mice and Presenilin-1 mutated fibroblasts, that the use of Ubisol-Q_10_ exerted the resumption of autophagy and presented implications for the inhibition of senescence and neuroprotection [100]. However, it remains unclear if the effects of these new formulations over the classical CoQ_10_ might be attributable to its increased solubility or to the ability to reach the brain through the BBB, so basic research in this sense should be carried out. Komaki et al. in 2019 used a rat model to study the neuroprotective role of CoQ_10_ on long-term potentiation, which measures the synaptic plasticity which is altered in AD due to β-amyloid deposits. They demonstrated that CoQ_10_ supplementation increases long-term potentiation in both intact and β-amyloid-injected rats. Furthermore, CoQ_10_ reversed the increases in serum MDA levels and total oxidative stress, indicating that CoQ_10_ treatment changed the oxidant/antioxidant balance in favour of antioxidants [101].

Recent reports have proved more promising regarding the use of CoQ_10_ in the amelioration of oxidative stress and mitochondrial dysfunction that seem to be beyond the pathophysiological events occurring in AD. However, more studies and specifically clinical trials are needed to demonstrate these effects in humans, where the use of new water-soluble formulation could play a prominent role.

### 9.2. Parkinson’s Disease

Parkinson’s disease (PD) is a chronic progressive neurodegenerative disorder which is mainly presented in older people. It is the second most prevalent neurodegenerative disorder after AD, affecting about 2–3% of people over 65 years [102]. PD is characterized by tremors, bradykinesia, postural, and gait disturbance. The key pathologic features of PD are striatal dopamine depletion, because of neural death in the Substantia Nigra, and the presence of intracellular inclusions such as Lewy bodies [103]. Regarding our knowledge of PD pathophysiology, there are multiple lines of evidence pointing out the role of mitochondrial damage and oxidative stress as major contributors to the pathogenesis. On the basis of these findings, we can find a significant decrease in the complex I activity of the electron transport chain, which is accompanied by decreasing levels of CoQ_10_. These led to CoQ_10_ being considered a potential therapeutic tool in the treatment of PD, due to both its function in the electron transport chain in the mitochondria and its potent antioxidant activity. However, due to its poor bioavailability, despite the promising results after in vitro assays, it has failed to demonstrate a beneficial effect in its use in PD patients. Recent investigation with novel formulations such as Ubisol-Q_10_, a water-soluble formula of CoQ_10_ mentioned above for its use in animal models of AD, has also been used in experimental animal models to evaluate its efficacy in PD. In this context, Muthukumaran et al. reported that Ubisol-Q_10_ effectively blocked neurodegenerative progression in a rat model of paraquat-induced neurodegeneration at acceptable daily doses of 12 mg/kg/day [104]. Sikorska et al. also demonstrated the effect of Ubisol-Q_10_ in blocking the neurodegenerative progression in a 1-methyl-4-phenyl-1,2,3,6-tetrahydropyridine mouse model by protecting the survival of dopaminergic cells with a robust astrocyte activation in the brain parenchyma, indicating the role of astroglia in this neuroprotection. However, they observed that disruption in the treatment stop the neuroprotection [105]. More recently, Onaolapo et al. reported benefits of CoQ_10_ supplementation in a rodent model of a chemically induced Parkinsonian disorder, reducing catalepsy, increasing dopamine, and reducing oxidative stress [106]. According to these studies, the idea that although CoQ_10_ seems to show promise in the treatment of PD, clinical trials are needed to demonstrate its potential effect, as well as the use or investigation of novel, more soluble formulations of this compound. 

### 9.3. Multiple System Atrophy

Multiple system atrophy (MSA) is a progressive neurodegenerative disease characterized by autonomic failure in addition to various combinations of parkinsonism, cerebellar ataxia, and pyramidal dysfunction [107]. The molecular basis underling the pathogenesis of MSA remains unknown. At the tissue level, MSA is characterized by the development of α-synuclein aggregates in the cytoplasm (glial cytoplasmic inclusions), primarily in oligodendrocytes, playing an important role in the pathogenic cascade leading to MSA [108]. The brain regions affected in MSA patients present expression of apoptotic-related proteins, which suggests an apoptosis-induction of neurons in these patients [109]. Regarding the aetiology of the disease, it has been suggested that there is both an environmental and a genetic predisposition. Whole-genome sequence analysis along with linkage analysis has shown homozygous or compound heterozygous mutations in *COQ2* in two of the six multiplex Japanese families with MSA [110]. *COQ2* encodes one of the enzymes involved in the biosynthesis pathway of CoQ_10_. In addition, total CoQ_10_ levels in frozen brain tissues and lymphoblastoid cell lines derived from MSA patients were lower than those from control subjects. These facts suggest the possible effect that CoQ_10_ supplementation could exert in these patients. However, little scientific evidence is available to prove the benefits of this treatment. Mitsui et al. reported a three-year follow-up with a high dose of 1200 mg/day of ubiquinol supplementation in a case of familial MSA with compound heterozygous *COQ2* mutations [111]. Despite the fact that the administration of high doses of ubiquinol leads to a substantial increase in the total CoQ_10_ levels in plasma, peripheral blood mononuclear cells, and even cerebellar spinal fluid, the first study reported that they did not detect any obvious neurological improvements, as determined by the rating scales. The authors suggest that this failure could be due to the advanced stage of neurodegeneration of the patients. In addition, the study suggested an improvement in the mitochondrial oxidative metabolism that could potentially alter the natural history of MSA progression when applied in the early stage of the disease. More recently, Nakamoto et al. demonstrated, in neurons differentiated from iPSCs derived from MSA patients, the existing genotype–phenotype causal relationship between *COQ2* mutations and the mitochondrial respiratory dysfunction observed in the disease. This fact showed the potential therapeutic use of CoQ_10_ for MSA patients with a *COQ2* mutation. In addition, the authors observed that in MSA, patient-derived neurons without a *COQ2* mutation also showed some decrease in mitochondrial respiratory functions, suggesting the potential use of supplementation even in this sort of patient [112]. It is particularly notable that in the case of MSA, the potential effect of CoQ_10_ supplementation might derive from the restoration of the endogenous levels, which could ameliorate the mitochondrial function and better control oxidative stress.

## 10. Conclusions

CoQ_10_ has been extensively studied since it was first described in 1955. Multiple functions have been unravelled during that time which contribute to our current knowledge of this molecule. However, the most prominent and relevant function is its antioxidant capacity. As an antioxidant, CoQ_10_ has demonstrated to be potentially used as a treatment in diseases which have oxidative stress as a hallmark in their aetiologies. The beneficial effects of CoQ_10_ have been reported in the treatment of chronic diseases. However, a consensus needs to be reached about the optimal dose for its therapeutic use with different diseases, since discrepancies in its effects are observed between different studies. On the other hand, the existence of various formulations, from the use of ubiquinol or ubiquinone to new analogues such as Ubisol-Q_10_ or Qter^®^, which vary in their bioavailability and effectiveness, makes it difficult to compare studies and reach a clear conclusion on the clinical use of CoQ_10_. A major effort must therefore be made in order to demonstrate its beneficial effects in clinical trials, which might allow CoQ_10_ to be implemented in clinical practice. 

## Abbreviations

AST	Aspartate aminotransferase
CAD	Coronary artery disease
CKD	Chronic kidney disease
COPD	Chronic obstructive pulmonary disease
CoQ10	Coenzyme Q10
CRP	C-reactive protein
eNOS	Endothelial nitric oxide synthase
EPCs	Endothelial progenitor cells
ESRD	End-stage renal disease
FMD	Flow-mediated dilation
GGT	Gamma-glutamyl transpeptidase
HF	Heart failure
HOMA-IR	Homeostatic model assessment of insulin resistance
hs-CRP	High-sensitivity C-reactive protein
Lp(a)	Lipoprotein (a)
MACE	Major adverse cardiac events
MitoQ	Mitochondrial-targeting antioxidant
MSA	Multiple system atrophy
NAFLD	Non-alcoholic fatty liver disease
NFκB	Nuclear factor kappa B
NMD	Nitroglycerin-mediated dilation
PPAR-γ	Peroxisome proliferator-activated receptor-gamma
RCTs	Randomized clinical trials
ROS	Reactive oxygen species
SQOR	Sulfide:quinone oxidorreductase
TAC	Total antioxidant capacity
TG	Triglyceride
